# A 3D Stable Metal–Organic Framework for Highly Efficient Adsorption and Removal of Drug Contaminants from Water

**DOI:** 10.3390/polym10020209

**Published:** 2018-02-22

**Authors:** Zhidong Luo, Shuran Fan, Jianqiang Liu, Weicong Liu, Xin Shen, Chuangpeng Wu, Yijia Huang, Gaoxiang Huang, Hui Huang, Mingbin Zheng

**Affiliations:** Dongguan Key Laboratory of Drug Design and Formulation Technology, Key Laboratory of Research and Development of New Medical Materials of Guangdong Medical University, School of Pharmacy, Guangdong Medical University, Dongguan 523808, China; luozhidong06@126.com (Z.L.); ShuranFan@163.com (S.F.); gdmulwc@126.com (W.L.); ShenXin_79@163.com (X.S.); 13537153056@163.com (C.W.); h173923790@163.com (Y.H.); gaoxianghuang2056@126.com (G.H.); hh13717432121@163.com (H.H.)

**Keywords:** metal–organic framework, drug contaminant, adsorption

## Abstract

We herein selected a 3D metal–organic framework decorated with carboxylate groups as an adsorbent to remove the pharmaceutical molecules of diclofenac sodium and chlorpromazine hydrochloride from water. The experiment aimed at exploring the effect factors of initial concentration, equilibrium time, temperature, pH and adsorbent dosage on the adsorption process. The adsorption uptake rate of the diclofenac sodium is much higher than that of the chlorpromazine hydrochloride. This paper presents the high adsorption capacity of diclofenac sodium, in which porous MOFs are used for the removal of drug contaminants from water. According to linear fitting with adsorption isotherm equation and kinetic equations, diclofenac sodium conforms to the Langmuir model and pseudo-first-order kinetic equation, while chlorpromazine hydrochloride accords with the Temkin model and pseudo-second-order kinetic equation. The results of the study indicate that the title compound could be a promising hybrid material for removing diclofenac sodium and chlorpromazine hydrochloride from wastewater.

## 1. Introduction

Pharmaceuticals and personal care products (PPCPs), as a new type of emerging pollutant in living environment, has drawn great attention owing to the potential hazard [1,2,3,4,5]. Usually, PPCPs contain active drugs, such as analgesics, antibiotics, anti-inflammatory drugs, and lipid regulators, and auxiliary materials, such as plasticizer, essence, emulgators, cosolvent, preservatives, etc. All of them must be detected and removed from the environment since these unwanted accumulations induce several health problems [3,5,6,7,8,9]. It has been reported that there are three main environmental pollution sources for PPCPs [2,10,11]: (1) PPCPs are discharged into the urban drainage system in the form of prototypes or metabolites through urine and feces from the body of human and livestock; (2) pharmacies and hospitals also are an important source that release pharmaceuticals into water bodies when they are used; and (3) in pharmaceutical factories, a variety of drugs and their byproducts in water are released with industrial effluent discharge.

Even though the concentrations of PPCPs in water are generally below the therapeutic dose [9], there is still enormous risk to environment and human health [8,12,13,14,15,16,17,18]. With the heavy use of antibiotics and the increasing antibiotics concentrations in water, bacteria in the water sample form resistance to drug by long-term exposure [19,20]. Meanwhile, when humans consume contaminated water over the long term, it will change the structure of microflora in the digestive tract and affect the body immunity [21]. Low concentrations of hormones drug in water environment would interfere with the normal hormone level of the aquatic animal and then change physiological function [8]. The worst situation could change biological community structure and reduce the functional diversity of aquatic communities. Eventually, aquatic ecosystems are destroyed and some species disappear [13,22,23]. The metabolic progress occurs slowly so that it cannot counteract the continuous discharge of drug contaminant in human activity. Consequently, even though drugs are not a long-lasting pollutant, it still presents a “false persistence” in water environment showing serious threat to ecological system and human health [12,13]. 

As for the problem of drug contaminant, environmentalists and scientists devote themselves to finding technology to purify contaminated water. Presently, adsorption technology, advanced oxidation processes (AOPs) [24], biological technologies, separation processes, and multiple-treatment processes are used to remove PPCPs from water [25,26]. Many materials such as mesoporous clay materials, zeolites, carbonaceous materials, and biosorbents have been developed as sorbents to remove PPCPs from water [12,25]. Metal–organic frameworks (MOFs), also known as coordination polymers or coordination networks, are crystalline materials built from metal ions or clusters bridged by organic linkers to form different dimensions [27,28,29,30,31,32,33,34,35,36,37,38,39]. These hybrid materials exhibit various properties, e.g., high relative surface area, order porous structure, magnetism and luminescence, and are hot spot in material field [30,31,32,33,34,35,36,37,38,39]. Until now, thousands of MOFs have been synthesized and applied in separations [40], gas store [41,42], drug delivery [40], biological imaging [43,44,45], and sensing [46,47]. At the same time they are studied as absorbent for removing dye [48,49], metal ion, drug contaminant [50,51,52,53,54,55,56,57] and organic solvent from water. With this in mind, a porous Cu-based MOF based on a pentacarboxylate ligand 2,5-bis(3′,5′-dicarboxylphenyl)-benzoic acid (H_5_L) [49], namely, [(CH_3_)_2_NH_2_]{[Cu_2_(L)·(H_2_O)_2_]·xsolvent}*_n_* (**1**), has been selected as a sorbent to remove diclofenac sodium and chlorpromazine hydrochloride from the aqueous solution. Compound **1** exhibits excellent gas adsorption capacity taking advantage of large permanent porosity. From the practical application perspective, the choice of **1** to remove contaminant drugs was based on the following features associated with this system: (a) **1** has relatively large surface area (about 1919.2 m^2^·g^−1^), which may enlarge the drug adsorption capacity; (b) similar to NOTT-101, it shows good water resistance; and (c) the uncoordinated −COO^−^ groups may contribute to drug selectivity. Moreover, the pharmaceutical molecules of diclofenac sodium and chlorpromazine hydrochloride are the most common health contaminants in PPCPs [12], and **1** exhibits high uptake rates as well as selective adsorption of diclofenac sodium in aqueous solution.

## 2. Experiments

### 2.1. Materials and Methods

Copper nitrate hydrate (99.5%), *N,N*-dimethylformamide (DMF, 99.5%), Ethanol absolute (99.5%) and Methanol (99.5%) were obtained from Tianjin Damao Chemical reagent Factory, Tianjin, China. 2,5-bis(3′,5′-dicarboxylphenyl)-benzoic acid (H_5_L, 98%) was obtained from Jinan Henghua technology Co. Ltd., Jinan, China. Chlorpromazine hydrochloride (98%, pk_a_ = 9.3) and diclofenac sodium (99%, pk_a_ = 4) were obtained from Shanghai Macklin Biochemical Co., Ltd., Shanghai, China. All chemicals were used as purchased without further purification. IR spectra were recorded on a WQF-510A FT-IR spectrometer (Beijing Beifen-Ruili Analytical Instrument Group Co., Ltd., Beijing, China) in the range of 500–4000 cm^−1^ using the KBr disc technique. Thermogravimetric analysis (TGA) was performed on a computer-controlled HCT-2 thermogravimetric analyzer (Beijing Hengjiu scientific instrument factory, Beijing, China).

### 2.2. Syntheses of ***1***

The preparation of **1** was based on the method reported by Liu [49] with some improvement. Copper nitrate hydrate (2.7 g, 0.011 mol) and H_5_L (0.9 g, 0.002 mol) was dissolved in a solution containing DMF (90 mL) and water (90 mL), and then 20 mL fluoroboric acid was added into the mixture with stirring. Then, 5 mL of the mixed solution in a glass bottle was heated at 95 °C for 1 day. The pale-blue crystals were separated. The obtained crystals were washed two times using ethanol. Caution: There is the chance of evaporation loss of water. 

The X-ray powder diffraction patterns of samples were used to check the phase purity and stability of **1** at room temperature. The experimental patterns are in good agreement with the simulated ones (Figure S1), which clearly indicates the high purity of the product.

### 2.3. Batch Adsorption Studies

Before the adsorption experiment, **1** was activated through immersing in methanol for 3 days and was then dried at 100 °C overnight [49]. Adsorption experiment on **1** was studied with two stock solution of chlorpromazine hydrochloride (1.5 g/L) and diclofenac sodium (1.5 g/L) prepared by dissolving each drug (1.5 g) into 1 L deionized water. Lower concentrations of the two drugs were obtained by successive dilution of the stock solution with deionized water. The calibration curves for the two drugs were plotted from the absorbance of the prepared drugs standard solutions. Twenty milliliters of different concentrations of drug solution and 10 mg adsorbents were placed in vial and then the vial was sealed. After that, the vial was shaken in an incubator shaker at a constant speed of 200 rpm at different temperature for a different time. The adsorbent materials were thereafter centrifuged and the drug concentration in the filtrates was determined using PAMADA UV-650 UV–Vis spectrophotometer for diclofenac sodium (λ_max_ = 274 nm) and for chlorpromazine hydrochloride (λ_max_ = 253 nm). The amount of drug adsorbed onto activated **1** was obtained by the expression.

(1) $$q_{t}=\frac{\left(C_{0}-C_{t}\right)V}{W}$$

where *q_t_* is the adsorption capacity of drug (mg/mg) at time *t* min, *C*_0_ and *C_t_* are the initial concentrations and certain concentration at time *t* of drug (mg/L), *V* is the volume of the solution of the adsorbate in vial (L), and *W* is the amount of adsorbent (mg). Based on the structural feature of the two drugs, the full measurement data concluded that the pH of diclofenac sodium solution was adjusted to 6.5–10.5 and the pH of chlorpromazine hydrochloride solution was adjusted as 3.5–6.5.

## 3. Result and Discussion

### 3.1. Structural Feature and Stability

**Figure 1:**
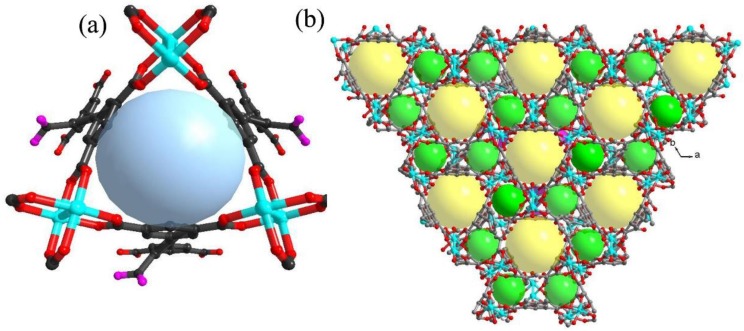
(**a**) View of the paddlewheel SBU (the uncoordinated –COO^−^ groups are marked as pink); and (**b**) view of the two cages which are tuned by the ligands (yellow ball represents large cage: 22 Å and green ball represents small cage: 10 Å).

Polymer {[Cu_2_(L)·(H_2_O)_2_] (**1**.xsolvent)} has a 3D NbO-type topological network, which has a basic structure similar to NOTT-101 [42]. The Cu(II) centers are bridged by four carboxylate groups to form a paddlewheel SBU (Figure 1a), which are further connected by L ligands to build a 3D porous network having two types of cages (Figure 1b) [49]. The authors have confirmed that the title compound can be stabilized under humid environment [49]. The great stability of **1** guarantees it retains its structure during the function study and thus establishes an excellent basis for its further application. This is also confirmed by TGA. Above 225 °C, **1** starts to decompose (Figure S2). The IR spectra of as-synthesized **1**, chlorpromazine hydrochloride, diclofenac sodium and their adsorbed states are shown in Figure S3. The bands of ν_as_(COO) and ν_s_(COO) were also observed, indicating that the –COOH groups in **1** are deprotonated. After the chlorpromazine hydrochloride or diclofenac sodium adsorption and activation, the ν_as_(COO) mode at 1620 cm^−1^ would blue-shift by 10–20 cm^−1^, while no obvious drift was observed for the weaker ν_s_(COO) band at 1442 cm^−1^. The transformation of the –COO^−^ vibration mode is due to the electrostatic interactions between the framework and drug molecules.

### 3.2. Adsorption Studies

To investigate the adsorption ability of **1** for chlorpromazine hydrochloride and diclofenac sodium, several factors were analyzed. The factors include initial concentration, contact time, temperature, solution pH and adsorbent dosage (all measurements are performed at pH 7.0). At the same time, the best value of the previous factor would be considered the optimized condition. Finally, the specific desorption process was obtained in sodium chloride solution at concentration of 10 mg/mL.

#### 3.2.1. Effect of Initial Concentration

**Figure 2:**
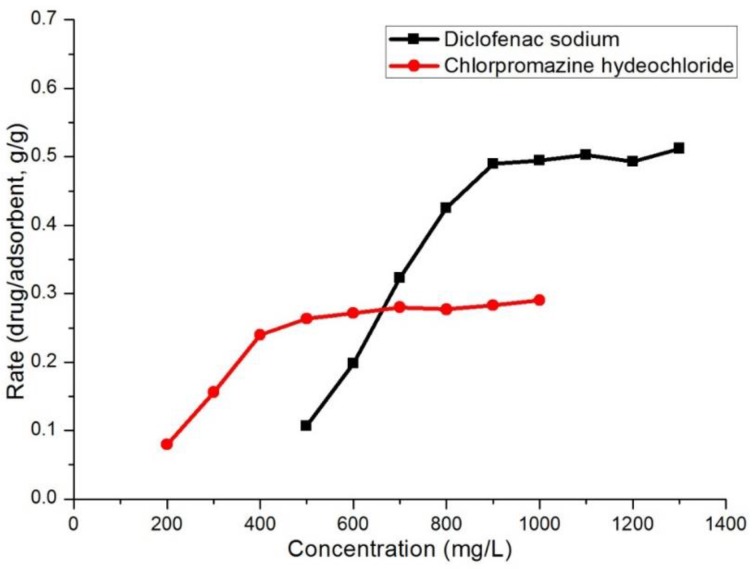
View of the adsorption capacity rate of chlorpromazine hydrochloride and diclofenac sodium.

The influence of drug initial concentration on the adsorption process is shown in Figure 2. The experiment was carried out at 25 °C for 10 h through adding 10 mg adsorbent to 20 mL aqueous solution of drug at different concentration (200–1000 mg/L for chlorpromazine hydrochloride, and 500–1300 mg/L for diclofenac sodium). The adsorption of diclofenac sodium and chlorpromazine hydrochloride increased with the increase of drug concentration. When the concentration was 700 mg/L, the adsorption capacity does not rise higher for chlorpromazine hydrochloride. However, saturation of adsorption capacity for diclofenac sodium takes place when the drug concentration is 900 mg/L. Moreover, equilibrium adsorption capacity rate to adsorbent (*g*/*g*) for diclofenac sodium is nearly 0.5, while chlorpromazine hydrochloride is close to 0.3. The color of adsorbent also clearly changed (Figure S4). The different results might be caused by structural nature of the two drugs. The title MOF has a negatively charged carbonyl group in the backbone (the charge was balanced by dimethylamine ion), making the framework electronegative. The chlorpromazine is positively charged so that the chlorpromazine is adsorbed to the framework by electrostatic interactions (Scheme S1). As for the diclofenac sodium, the free sodium ions may be easily adsorbed into the host framework, resulting in diclofenac anion also adsorbing into the adsorbent by electrostatic effect. Nevertheless, chlorpromazine molecular skeleton is bigger than that of the diclofenac sodium (Figure S5), so it would take up more space in the framework. Furthermore, the π–π stacking interactions between the aromatic rings of drugs and the host MOF **1** might contribute to its excellent adsorption performance towards diclofenac anion. The previously reported adsorption capacity of MOFs is summarized in Table 1. Comparing to the reported MOFs, the title MOF shows higher adsorption capacity of diclofenac sodium.

#### 3.2.2. Effect of Time 

**Figure 3:**
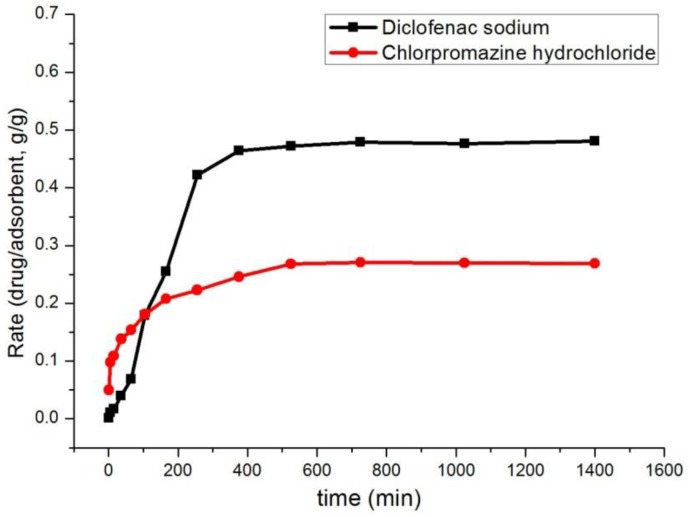
The effect of contact time on the adsorption of the chlorpromazine hydrochloride and diclofenac sodium.

The experiment was carried out at 25 °C with 900 mg/L solution of diclofenac sodium or 700 mg/L solution of chlorpromazine hydrochloride and 10 mg of adsorbents. To determine the equilibrium time of adsorption progress, the adsorption of chlorpromazine hydrochloride and diclofenac sodium on the adsorbents was studied over a contact period of 0–1400 min. As shown in Figure 3, the chlorpromazine hydrochloride maintained a relatively rapid adsorption for 200 min with a rate of 20%, while the adsorption rate was relatively slow between 200 and 500 min, and the adsorption equilibrium (≈28%) was reached at 500 min. The profile of this adsorption progress is mainly caused by the mechanism of electrostatic interaction. In contrast, the diclofenac sodium presented a fast and stable adsorption with an adsorption rate of 42% in 250 min; after that, it slowed from 250 min to 700 min and reached adsorption equilibrium with 48% adsorption rate. 

#### 3.2.3. Effect of Temperature on Adsorption

**Figure 4:**
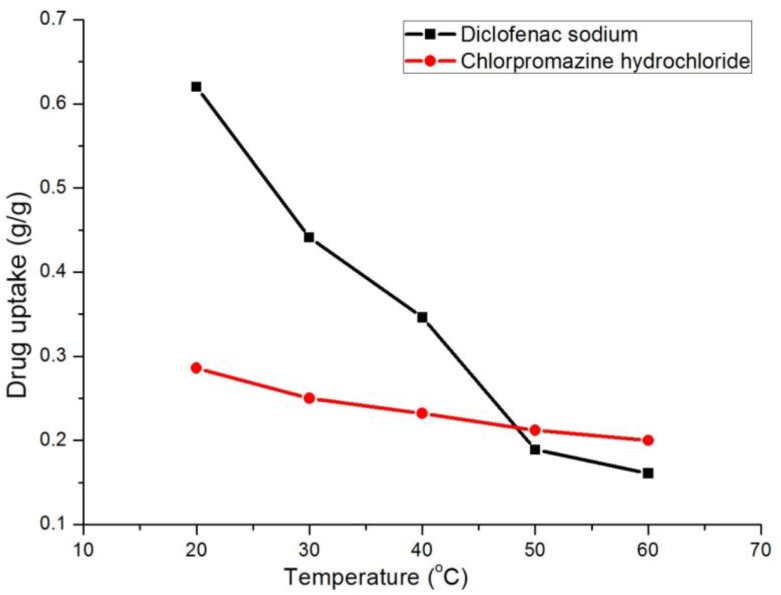
The effect of temperature on the adsorption of chlorpromazine hydrochloride and diclofenac sodium.

Temperature was an important factor on the adsorption progress, thus we studied the adsorption effect of drugs in the range 20–60 °C. Ten milligrams of sorbent was placed in 20 mL of 900 mg/L diclofenac sodium or 700 mg/L chlorpromazine hydrochloride aqueous solution. The results indicated that the adsorption effect of the two drugs exhibited evident decreases with increasing temperature (Figure 4). It can be explained that the adsorption process is an exothermic process. When temperature increased, the adsorption process is suppressed. Thus, the desorption is faster, making the free drug molecules increase. The results also show that temperature has different influence on the adsorption of diclofenac sodium and chlorpromazine hydrochloride. When the temperature was 20 °C, adsorbing capacity was 62% for diclofenac sodium and 28% for chlorpromazine hydrochloride. At 60 °C, it decreased to 16% for diclofenac sodium and 20% for chlorpromazine hydrochloride.

#### 3.2.4. Effect of pH on Drug Adsorption

**Figure 5:**
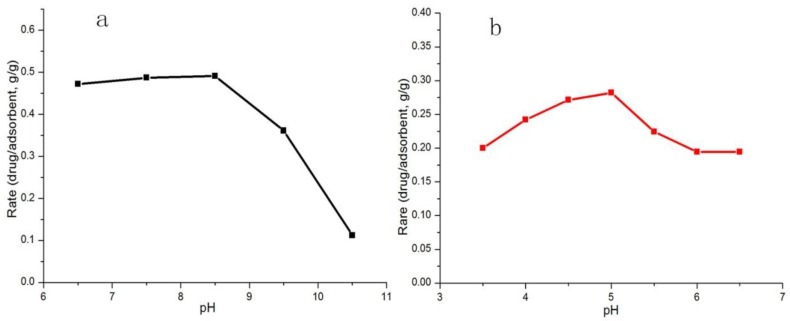
The effect of pH on the adsorption of: diclofenac sodium (**a**); and chlorpromazine hydrochloride (**b**).

To explore the effect of pH value on the adsorption process, the adsorption process was tested at different pH values at 25 °C and the adsorption capacity was determined. Because diclofenac sodium solution in low pH (≤6) will be easily precipitated, the pH of diclofenac sodium solution was adjusted to 6.5–10.5. On the contrary, chlorpromazine hydrochloride solution will be precipitated when pH is greater than 7. Thus, the pH of chlorpromazine hydrochloride solution was adjusted to 3.5–6.5. The drug solutions were regulated by 0.01 mol/L of sodium hydroxide solution and 0.01 mol/L hydrochloric acid. As shown in Figure 5, the adsorption of diclofenac sodium did not change significantly with pH increasing from 6.5 to 8.5, but a significant decrease appeared as pH increased from 8.5 to 10.5. The maximum adsorption amount of diclofenac sodium was 49% when pH was 8.5. For chlorpromazine hydrochloride, the maximum adsorption amount was 27% when the pH was 5. Thus, the results indicated peracidity or parlkaline would reduce the adsorption capacity. 

#### 3.2.5. The Effect of Adsorbent Dosage

**Figure 6:**
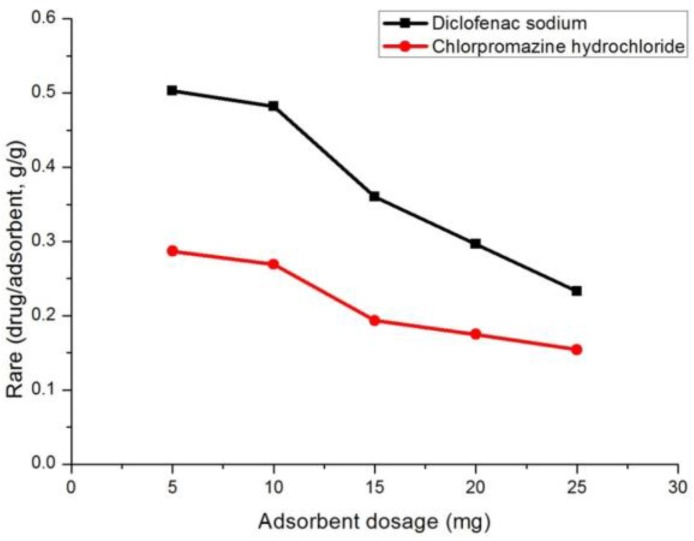
The effect of adsorbent dosage on the adsorption of the two drugs.

The effect of adsorbent dosage on the adsorption of the chlorpromazine hydrochloride and diclofenac sodium was also studied (Figure 6). The experiment was carried out in the range 5–25 mg of adsorbent dosage under room temperature. The results revealed that the adsorption capacity of chlorpromazine hydrochloride and diclofenac sodium decreased rapidly with increasing the mass of the adsorbent. Apparently, when the adsorbent dosage was more than 10 mg, the adsorbent still does not reach saturation. The drug contaminants removed from the solution will actually be increased.

#### 3.2.6. Desorption Process

**Figure 7:**
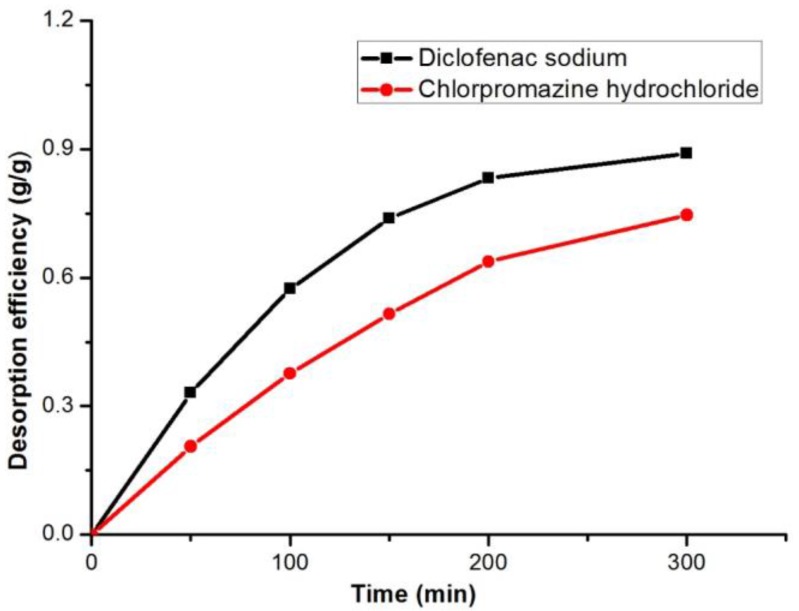
The desorption processes of incorporation of adsorbent and diclofenac sodium or chlorpromazine hydrochloride.

Usually, an ideal adsorbent can be recovered and recycled. The desorption experiment was performed using a few successive steps by exchanging the supernatant solution with fresh desorption solution (Figure 7). After 200 min of stirring, about 85% of diclofenac sodium can be desorbed from the adsorbent. Unfortunately, no more diclofenac sodium was released after 300 min. For chlorpromazine hydrochloride, the rate was even lower (about 60%) and it basically reached the equilibrium of desorption after stirring for 200 min. In general, the adsorbent in a certain extent could remove the adsorbate after adsorption saturation. 

### 3.3. Adsorption Isotherms

For a better understanding of the adsorption equilibrium data of the two drugs of diclofenac sodium and chlorpromazine hydrochloride, their behaviors were analyzed using the Langmuir, Freundlich and Temkin isotherm models. These isotherm models are given in linear forms [58] as:

(2) $$\frac{C_{\mathrm{eq}}}{q_{\mathrm{eq}}}=\frac{1}{K_{L}\cdot q_{\max}}+\frac{C_{\mathrm{eq}}}{q_{\max}}\;\left(\mathrm{Langmuir}\;\mathrm{model}\right)$$

(3) $$\log q_{\mathrm{eq}}=\log K_{F}+\frac{1}{n}\log C_{\mathrm{eq}}\;\left(\mathrm{Freundlich}\;\mathrm{model}\right)$$

(4) $$q_{\mathrm{eq}}=B\ln A_{T}+B\ln C_{\mathrm{eq}}\;\left(\mathrm{Temkin}\;\mathrm{model}\right)$$

(5) $$\ln q_{\mathrm{eq}}=\ln q_{\max}-\beta \varepsilon^{2}\;\left(\mathrm{Dubinin}-\mathrm{Radushkevich}\right)$$

where *q*_max_ is the maximum adsorption capacity (mg/mg), *q*_eq_ is the amount adsorbed per unit mass of the adsorbent at equilibrium (mg/mg), *C*_eq_ is the equilibrium concentration of adsorbate (mg/L), *n* is the intensity of the adsorption constant, *K*_F_ (mg/mg) is the adsorption capacity for Freundlich model, *K*_L_ (L/mg) is Langmuir constant relating to adsorption strength or intensity, *A*_T_ is Temkin isotherm equilibrium binding constant (L/g), *B* is the constant related to heat sorption (J/mol), β is the Dubinin–Raduskevich isotherm constant, and *ε* is the mean free energy (KJ/mol).

**Figure 8:**
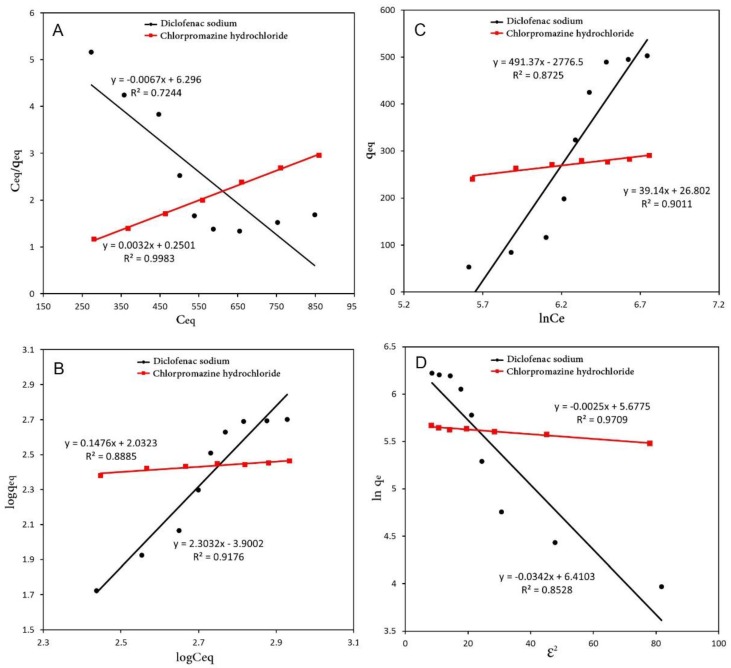
Langmuir (**A**); Freundlich (**B**); Temkin (**C**) and Dubinin-Radushkevich (**D**) isotherm linear plots for the adsorption of diclofenac sodium and chlorpromazine hydrochloride.

According to the linear fitting, the theoretical parameters for the isotherms were used in this study and their regression coefficient values are summarized in Table 2. In Figure 8, it can be seen that the adsorption progress of diclofenac sodium is more fitted to the Temkin model among the four isotherm model with the *R*^2^ = 0.9498, while chlorpromazine hydrochloride is confirmed with the Langmuir isotherms with the *R*^2^ = 0.9927. 

### 3.4. Adsorption Kinetics

To understand the adsorption kinetics, the pseudo-first-order and pseudo-second-order kinetic models were used to describe the adsorption kinetics of diclofenac sodium and chlorpromazine hydrochloride. The rates of adsorption were correlated to the amount of diclofenac sodium and chlorpromazine hydrochloride adsorbed at certain time “*t*”.

The pseudo-first-order rate equation is given as [59]:

(6) $$\ln(q_{\mathrm{eq}}-q_{t})=\ln q_{\mathrm{eq}}-k_{1}t$$

The pseudo-second-order rate equation is given by [60]:

(7) $$\frac{t}{q_{t}}=\frac{1}{k_{2}q_{\mathrm{eq}}^{2}}+\frac{1}{q_{\mathrm{eq}}}t$$

where *q_t_* is the amounts of diclofenac sodium or chlorpromazine hydrochloride adsorbed per unit mass of the adsorbent (mg·g^−1^) at time *t*; *q*_eq_ is the equilibrium amounts of diclofenac sodium or chlorpromazine hydrochloride; *k*_1_ is a pseudo-first-order kinetic constant expressed in min^−1^; and *k*_2_ is the pseudo-second-order rate constant given in (g·mg^−1^·min^−1^). 

**Figure 9:**
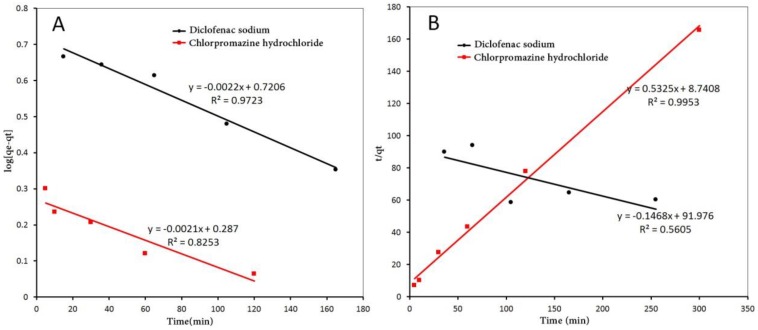
(**A**) The pseudo-first-order kinetic plot of chlorpromazine hydrochloride and diclofenac sodium; and (**B**) the pseudo-second-order kinetic plot of chlorpromazine hydrochloride and diclofenac sodium.

The results of linear fitting are presented in Figure 9 and the constants are listed in Table 3. These results could preliminary verify that the adsorption process of diclofenac sodium likely meets the pseudo-first-order kinetics with correlation coefficient value *R*^2^ = 0.9723. Compared to diclofenac, chlorpromazine hydrochloride accords with the pseudo-second-order kinetics with *R*^2^ = 0.9953. The calculated values of *k*_2_ or *k*_1_ for the adsorption of drugs on **1** is much higher than those reported for MOF-235 (2.28 × 10^−4^ g·mg^−1^·min^−1^) and ami-no-MIL-101(Al) (2.68 × 10^−3^ g·mg^−1^·min^−1^) [61]. This is mainly because the anionic framework has strong interactions with the drugs molecules. It is proven that **1** can quickly adsorb diclofenac than many adsorbents.

## 4. Conclusions

We have selected a hydrostable 3D MOF as an adsorbent to remove diclofenac sodium and chlorpromazine hydrochloride. It is found that diclofenac sodium exhibits faster rate of uptake than that of chlorpromazine hydrochloride. The adsorption capacity of **1** for diclofenac sodium was 900 mg/L at 293 K. The adsorption capacities of the two drugs for **1** would be reduced under peracidity or parlkaline condition. The comparison between the adsorption capacities of two drugs suggests that the drugs might involve a weak interaction between the uncoordinated –COO^−^ groups of host MOF **1** and drugs. The kinetics, adsorption isotherm and thermodynamics of drug adsorptions on **1** were also investigated in this study. The present study provides new insight into the design of MOFs for PPCPs adsorption applications.

## Figures and Tables

**Table 1:** The adsorption effect (*g*/*g*) of previously reported MOFs.

Adsorbent	Adsorbate	Adsorption Effect (*g*/*g*)	Reference
MIL-101	Dimetridazole	0.141	[55]
urea-MIL-101	Dimetridazole	0.185	[55]
urea-MIL-101	Metronidazole	0.188	[55]
UiO-66	Sulfachloropyradazine	0.417	[51]
ZIF-67-H_2_O	Sulfachloropyradazine	~0.028	[51]
ZIF-67-CH_3_OH	Sulfachloropyradazine	~0.030	[51]
YCM-101	Tetracycline	0.032	[56]
MIL-53(Fe)	Doxycycline	0.322	[53]
ZIF-8	Diclofenac sodium	0.320	[52]
ZIF-8	Ibuprofen	0.400	[52]
**1**	Diclofenac sodium	0.490	This work
**1**	Chlorpromazine hydrochloride	0.290	This work

**Table 2:** Isotherm parameters for the adsorption of diclofenac sodium and chlorpromazine hydrochloride.

Drug	Langmuir			Freundlich			Temkin			D-Radushkevich		
	*q*_max_	*K*_L_	*R*^2^	*K*_F_	*n*	*R*^2^	*B*_T_	*A*_T_	*R*^2^	*q*_max_	β	*R*^2^
Diclofenac sodium	−114.93	−967.00	0.7244	0.0003	0.4816	0.9176	294.7	0.0033	0.8725	315.35	−0.0289	0.9011
Chlorpromazine hydrochloride	333.33	0.0073	0.9983	69.1671	4.7259	0.8685	55.02	0.2164	0.9011	296.57	−0.0053	0.9709

**Table 3:** Pseudo-first-order and pseudo-second-order constants of chlorpromazine hydrochloride and diclofenac sodium.

Drug	Pseudo-First-Order		Pseudo-Second-Order	
	*k*_1_ (min^−1^)	*R*^2^	*k*_2_ (g·mg^−1^·min^−1^)	*R*^2^
diclofenac sodium	−0.0022	0.9723	−0.0016	0.5605
chlorpromazine hydrochloride	−0.0021	0.8253	0.0324	0.9953

## Supplementary Materials

The following are available online at http://www.mdpi.com/2073-4360/10/2/209/s1, Figure S1: XRD patterns of **1** simulated from X-ray crystal diffraction data and measured for the as-synthesized samples, after soaking/desorption in diclofenac sodium and chlorpromazine hydrochloride, respectively, Figure S2: View of the TGA, Figure S3: View of the IR, Figure S4: View of the color change before equilibrium adsorption capacity: (a) the as-synthesized samples; (b) after adsorb chlorpromazine; (c) after adsorb diclofenac sodium, Figure S5: The structure of diclofenac sodium (A) and chlorpromazine hydrochloride (B), Scheme S1: View of the dissociated state of chlorpromazine in aqueous solution.
