# Supplementary material for: A 3D Stable Metal–Organic Framework for Highly Efficient Adsorption and Removal of Drug Contaminants from Water
Source: Polymers (Basel). 2018 Feb 22;10(2):209. doi: 10.3390/polym10020209 (PMC6414845; doi:10.3390/polym10020209)
Supplement: Supplementary file 1 [file polymers-10-00209-s001.pdf]

A 3D stable metal-organic framework for highly efficient adsorption and removal of drug contaminants from water

Zhidong Luo[#], Shuran Fan[#], Jianqiang Liu*, Weicong Liu, Xin Shen, Chuangpeng Wu,

Yijia Huang, Gaoxiang Huang, Hui Huang, Mingbin Zheng*

Dongguan Key Laboratory of Drug Design and Formulation Technology, Key Laboratory of Research and Development of New Medical Materials of Guangdong Medical University, School of Pharmacy, Guangdong Medical University, Dongguan, 523808, P. R. China.

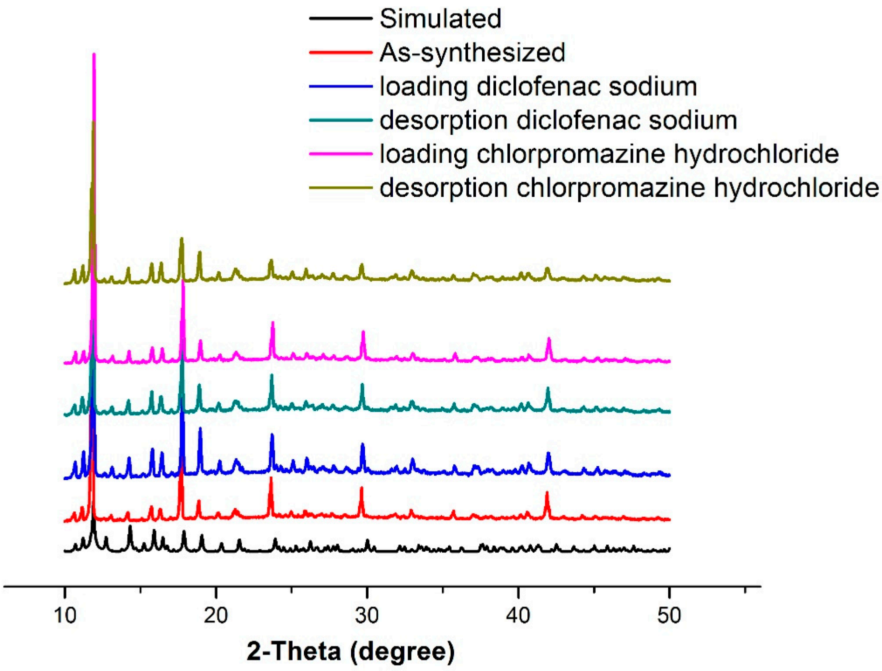

Fig. S1 XRD patterns of **1** simulated from X-ray crystal diffraction data and measured for the as-synthesized samples, after soaking/desorption in diclofenac sodium and chlorpromazine hydrochloride, respectively.

Thermogravimetric Analysis

Thermogravimetric Analysis was performed on a thermogravimetric analyzer HCT-2 (Beijing Hengjiu scientific instrument factory, china). 10 mg sample was weighted on crucible and heated from 25 to 800 °C at the speed of 10 °C /min with nitrogen atmosphere.

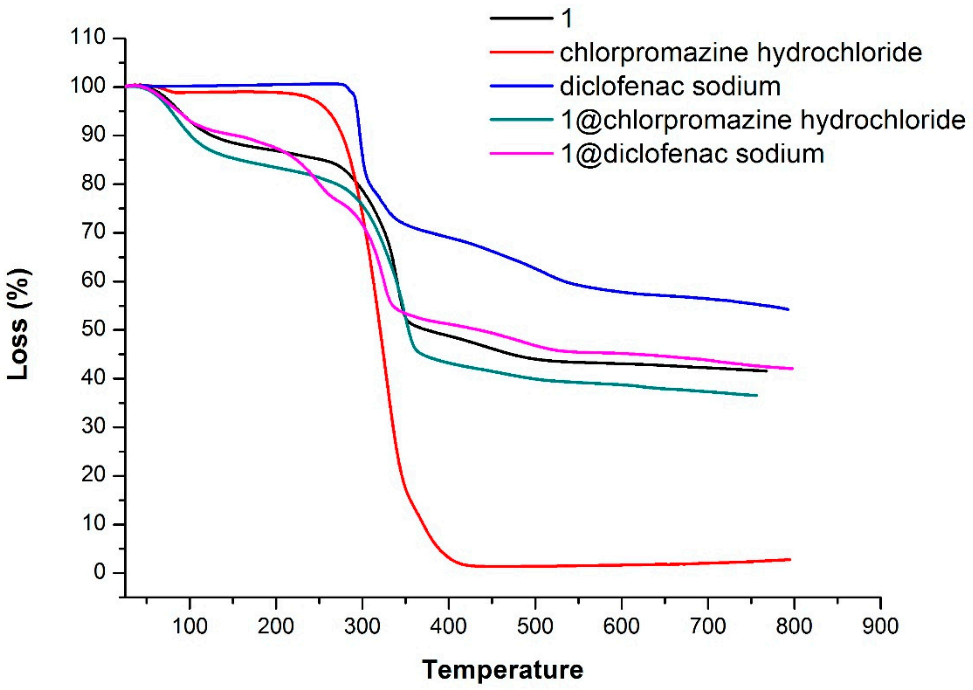

Fig. S2 view of the TGA.

The IR spectra of **1** exhibit the main characteristic absorption bands in the range of 1362 –1680 cm^{-1} mainly attributed to the asymmetric and symmetric stretching vibrations of the carboxylato groups. Δν[νas(COO)–νs(COO)] are 139 cm^{-1}, indicating coordination bidentated bridging modes of carboxylato groups to the central metal atom. The band at 3402 cm^{-1} is assigned to the stretching vibrations ν(OH) of water molecules. The bands at ca. 1545 cm^{-1} are assigned to the ν(C-N) absorption in the $(CH_3)_2NH_2$.

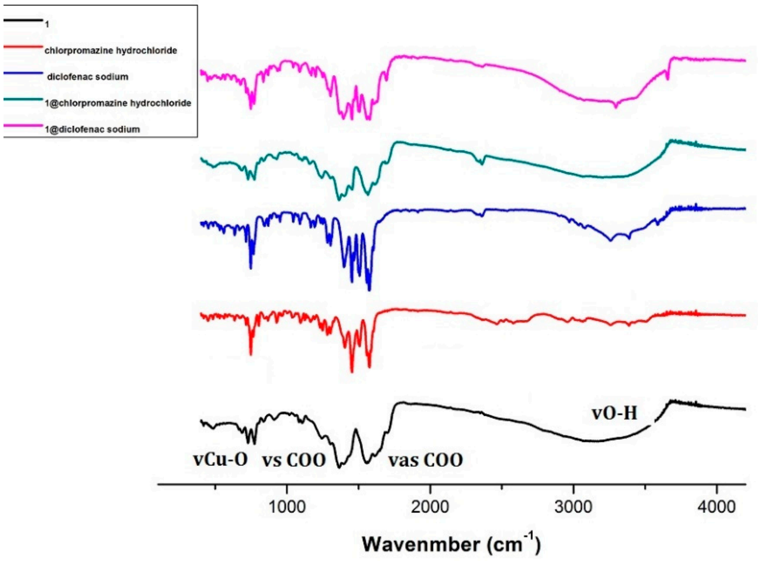

Fig. S3 view of the IR.

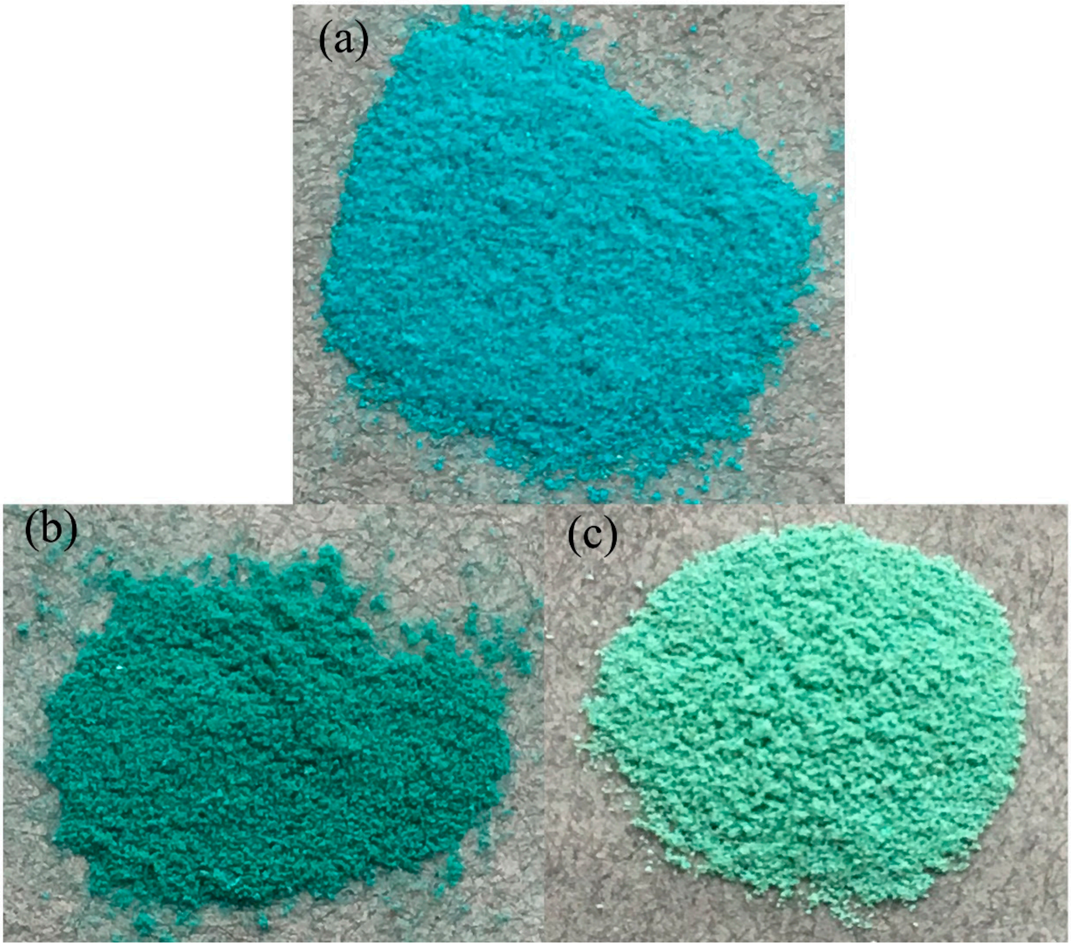

Fig. S4 view of the color change before equilibrium adsorption capacity: (a) the

as-synthesized samples; (b) after adsorb chlorpromazine; (c) after adsorb diclofenac sodium.

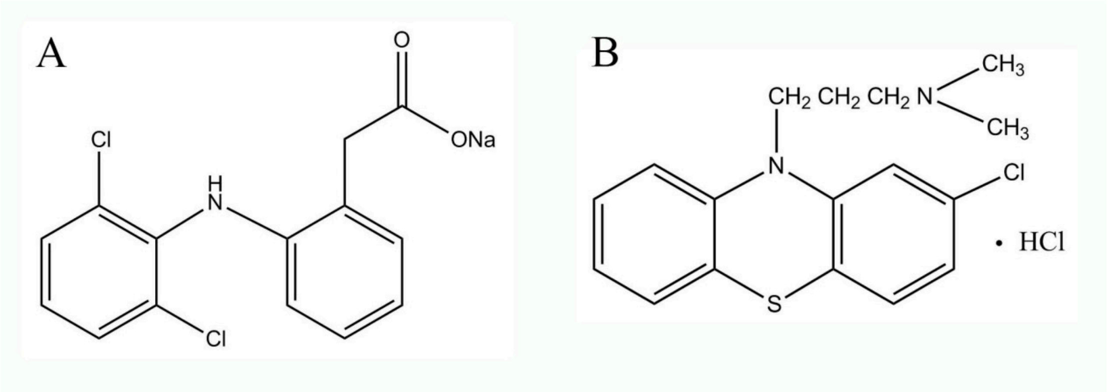

Fig.S5 The structure of diclofenac sodium (A) and chlorpromazine hydrochloride (B).

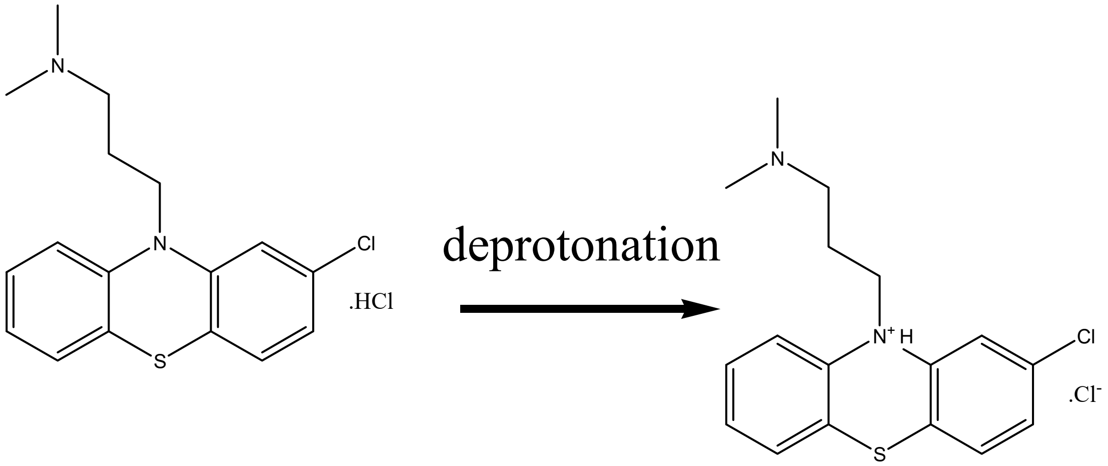

Scheme S1 view of the dissociated state of chlorpromazine in aqueous solution.
